# A longitudinal study on the correlation between faecal urease activity and incidence of nappy rash in infants

**DOI:** 10.3389/fped.2026.1803707

**Published:** 2026-05-25

**Authors:** Krystal A. Le Doaré, Vicky Hunt, Robyn Deeks, Amanda Vick, A. Toby. A. Jenkins

**Affiliations:** 1 Department of Chemistry, University of Bath, Bath, United Kingdom; 2 Department of Life Sciences, University of Bath, Bath, United Kingdom; 3 Westwood Nursery, University of Bath, Bath, United Kingdom

**Keywords:** ammonia, diaper dermatitis, faeces, nappy rash, urease

## Abstract

**Introduction:**

Nappy (diaper) rash is a common skin condition experienced by most infants at least once during the first 2 years of life. Despite its prevalence, there is some debate over its pathogenesis. The objective of this study was to investigate whether a correlation exists between urease activity in infant faeces and the observed incidence of nappy rash (diaper dermatitis). This was explored in a longitudinal observational study of six infants conducted at a university childcare facility over 9 months.

**Methods:**

Six babies who met the inclusion criteria and attended Westwood Nursery, University of Bath, were recruited to the study with consent provided by parents following favourable ethical approval from the NHS Regional Ethics Committee. Soiled nappies, donated up to twice weekly, were analysed for urease activity in faecal bacteria. At the same time, nursery staff recorded the skin condition of the nappy area for participating infants.

**Results:**

Chi-squared analysis revealed a clear statistical correlation between urease expression and observed nappy rash incidence, as well as between the absence of faecal urease and healthy skin (*P* = <0.0002).

**Discussion:**

Urease-expressing bacteria were first implicated in the pathogenesis of nappy rash in the early 20th century. This is the first study to demonstrate a population-level correlation between nappy rash and faecal enzyme activity, which can be understood in terms of a causal chain: Urease catalyses ammonia production, which directly damages skin barrier function and creates a pH environment in which secondary opportunistic microorganisms can grow at an enhanced rate. This, in turn, increases skin-damaging enzyme activity, leading to more severe nappy rash.

## Graphical Abstract

**Figure:**
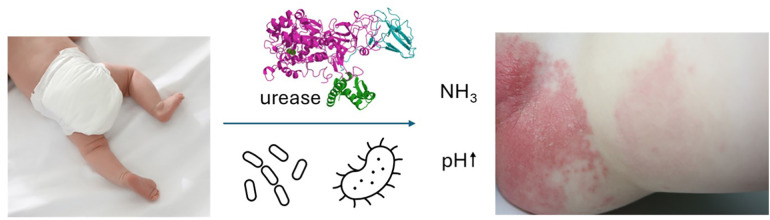

## 1 Introduction

Nappy rash, also known as diaper dermatitis, is a form of irritant contact dermatitis caused by prolonged exposure of skin to urine and faeces. It is estimated that 70% of infants experience at least once incidence of nappy rash during the first 24 months of their lives. Although not life-threatening, it causes significant pain and discomfort, resulting in reduced sleep and stress for both babies and their parents (1). Nappy rash typically presents as red and inflamed skin in the nappy area, with skin ulceration and obvious fungal infection observed in some cases. Nappy rash in incontinent adults is generally referred to as incontinence-associated dermatitis (IAD) and is arguably a more serious condition, as often observed in patients with poor mobility, pressure injury, and various co-morbidities, and can lead to serious tissue breakdown (2).

**Figure 1:**
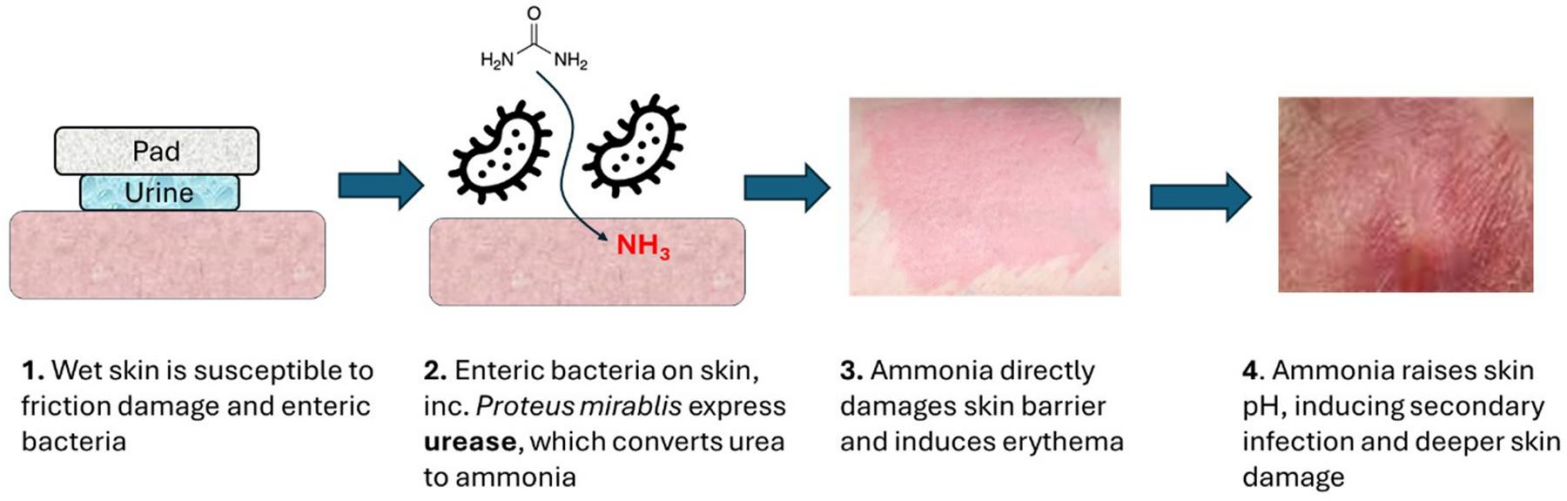
Proposed pathogenesis of nappy rash from early-stage skin redness caused by ammonia to later-stage opportunistic infection and skin breakdown, showing the central role of the enzyme urease.

The pathogenesis of nappy rash is complex. Human skin has not evolved to be in frequent contact with urine and faeces, exposing it to a complex ecology of pathogenic bacteria. Initially, these are enteric bacteria such as *Proteus mirabilis* and *Klebsiella* species, which express the enzyme urease. Urease converts urea found in urine and sweat into ammonia. Ammonia directly damages skin barrier function, creating visible erythema (Figure 1) (3, 4). Ammonia also increases skin pH, damaging the natural acid mantle of the stratum corneum and creating an environment in which the activities of faecal enzymes, including lipases, and proteolytic enzymes, such as trypsin, are enhanced, the growth rate of pathogenic commensal bacteria including *Staphylococcus aureus* is accelerated, and fungi such as *Candida albicans* are able to alter their morphology to a skin-invasive form, creating more severe skin damage.

The association of ammonia and nappy rash was first reported almost 100 years ago. In 1927, Cook and Keith described “an organism isolated in pure culture from the stools of more than 50 infants and older children involved in the splitting of urinary urea by a bacterium which infests the gluteal region and diaper from the feces,” which they named *Brevibacterium ammoniagenes* (5). In 1952, further bacteriological study of bacteria believed to be involved in nappy rash was undertaken by Brown et al. (6). They proposed that nappy rash took hold in two phases: initial skin injury-caused by ammonia expressing bacteria, followed by opportunistic infection with pathogens such as *Staphylococcus* species. However, this association between ammonia and nappy rash has been questioned. In 1977, a study by Leyden et al. attempted to measure the ammonia concentration in urine removed from the nappies of two groups of infants: those who had active dermatitis and those who did not. No statistical difference in ammonia concentration was measured (7). A subsequent paper by Berg in 1986, looking at the effect of urine and faeces on bald mice suggested that the pathogenic role of ammonia in nappy rash was via due to it increasing skin pH, and thus increasing the activity of skin damaging enzymes (8).

The putative role of urease-expressing bacteria and ammonia damage to skin is surprisingly absent from much of the recent literature. In a review from 2022 by Petek et al. on emerging links between infant microbiome and skin immunology in nappy rash, urease was not mentioned, and ammonia was only discussed in the context of how it raises skin pH and enhances the activity of faecal enzymes (9). Zheng expanded upon this idea and found that certain Phyla such as *Proteobacteria* and *Enterococcus—*which are both known to contain some urease-expressing species—showed significantly increased activity in infants with nappy rash compared with normal infant skin samples, correlating with our proposed mechanism (10).

Owen et al. compared the effects of sodium hydroxide and ammonia at equivalent pH values following application to the ventral forearms of adult humans. The study demonstrated that ammonia exerted a direct damaging effect on the skin, inducing erythema and substantially reducing stratum corneum barrier function, as measured by impedance spectroscopy, whereas sodium hydroxide produced no detectable effect (3). This finding raised important questions about the role of urease and ammonia in nappy rash. A 2023 paper by Kohta et al. reported a strong association between urease activity in genital skin swabs of incontinent stroke patients and the incidence of incontinence-associated rash (11). This supports an interesting observation as the effect of urease on skin leads to *in situ* ammonia production. Against this backdrop, the present study sought to address the following question: Is there a temporal correlation between urease expression from faecal bacteria and the incidence of nappy rash/IAD?

In this paper, we report a small longitudinal study of infants (age 6–18 months) at the University of Bath childcare facility, Westwood Nursery. Our start point was an observation: Given that babies' skin is constantly exposed to urine and faeces, why don't babies suffer from nappy rash all the time? The hypothesis being tested was that the urease expression levels of faecal bacteria to which babies are exposed are associated with incidences (or absence) of nappy rash. The rationale was that a possible association further supports the urease/ammonia hypothesis suggested by Owen et al. and the observations of adult IAD made by Kohta et al. (3, 11).

## 2 Methods

The study protocol and participant information sheet are included in the Electronic Supporting Information.

### 2.1 Study design

#### 2.1.1 Objectives

This longitudinal cohort observational study has the following primary objectives: (1) to examine the correlation between the faecal flora—specifically urease-expressing bacteria—of infants in the study and the incidence of nappy rash over the 8-month study period; and (2) to assess the recorded nappy rash incidence in infants and relative urease activity in their faeces over the 8-month period.

#### 2.1.2 Participant selection

Twelve infants were eligible for inclusion. Seven were initially recruited, but one infant was excluded due to insufficient collection of soiled nappies during nursery hours. Six infants were followed throughout the 8-month study. The five babies not included were scheduled to move to the next nursery room during the study period, making participation logistically challenging. Moreover, some parents declined consent.

#### 2.1.3 Consent and ethics

Parents of eligible babies were contacted by nursery staff and given a detailed patient information sheet (see ESI) before being asked to formally consent to enrolment of their child in the study. No payment or other incentive was offered to participating parents. Participating infants were given anonymous IDs, and the scientific team had no knowledge of the true identities of the babies or their parents. Ethical approval was granted by the NHS Regional Ethics committee (PR committee REC reference 23/NW/0390; IRAS project ID: 332482).

#### 2.1.4 Inclusion and exclusion criteria

Inclusion Criteria: Infants aged 6–18 months at study start; parental consent; infants wearing nappies (diapers) 24 h/day; and infants wearing disposable nappies.

Exclusion criteria: Infants whose parents did not consent; infants wearing reusable nappies; and infants whose parents' English language was insufficient to properly understand the study.

#### 2.1.5 Study methodology

**Figure 2:**
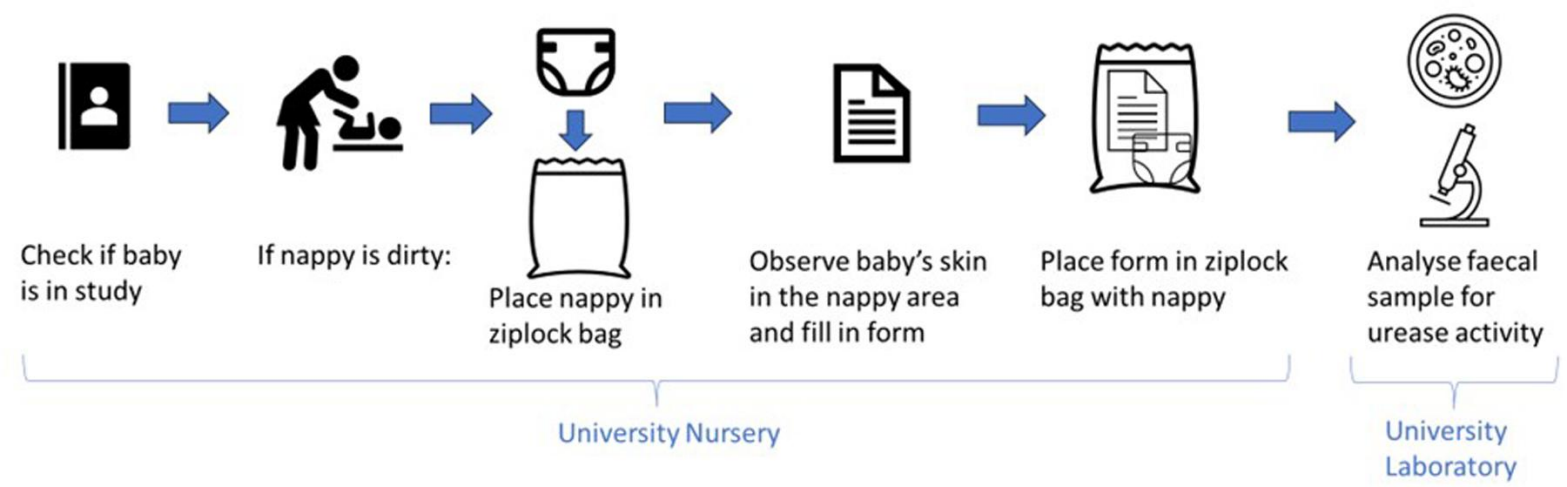
Schematic of study methodology, sample collection, and analysis of faecal samples.

Infants participating in the trial had their soiled (faecal) nappies placed in Ziploc bags labelled with their anonymous participant ID as part of their normal care routine. Nursery staff recorded the condition of the skin in the nappy area (see ESI Figure 1). The completed form was added to the Ziploc bag, and soiled nappies were collected by scientific staff at 17:00 on study days (Tuesdays and Thursdays). Faecal matter (ca. 2 g) was placed into Eppendorf tubes in the laboratory and flash-frozen in liquid nitrogen before being stored at −20°C until required. Eppendorf tubes were labelled with the infant ID number and collection date to ensure traceability of samples. Figure 2 illustrates the study methodology. The Participant Information Sheet and Study Protocol are provided in the ESI.

### 2.2 Sample processing

**Figure 3:**
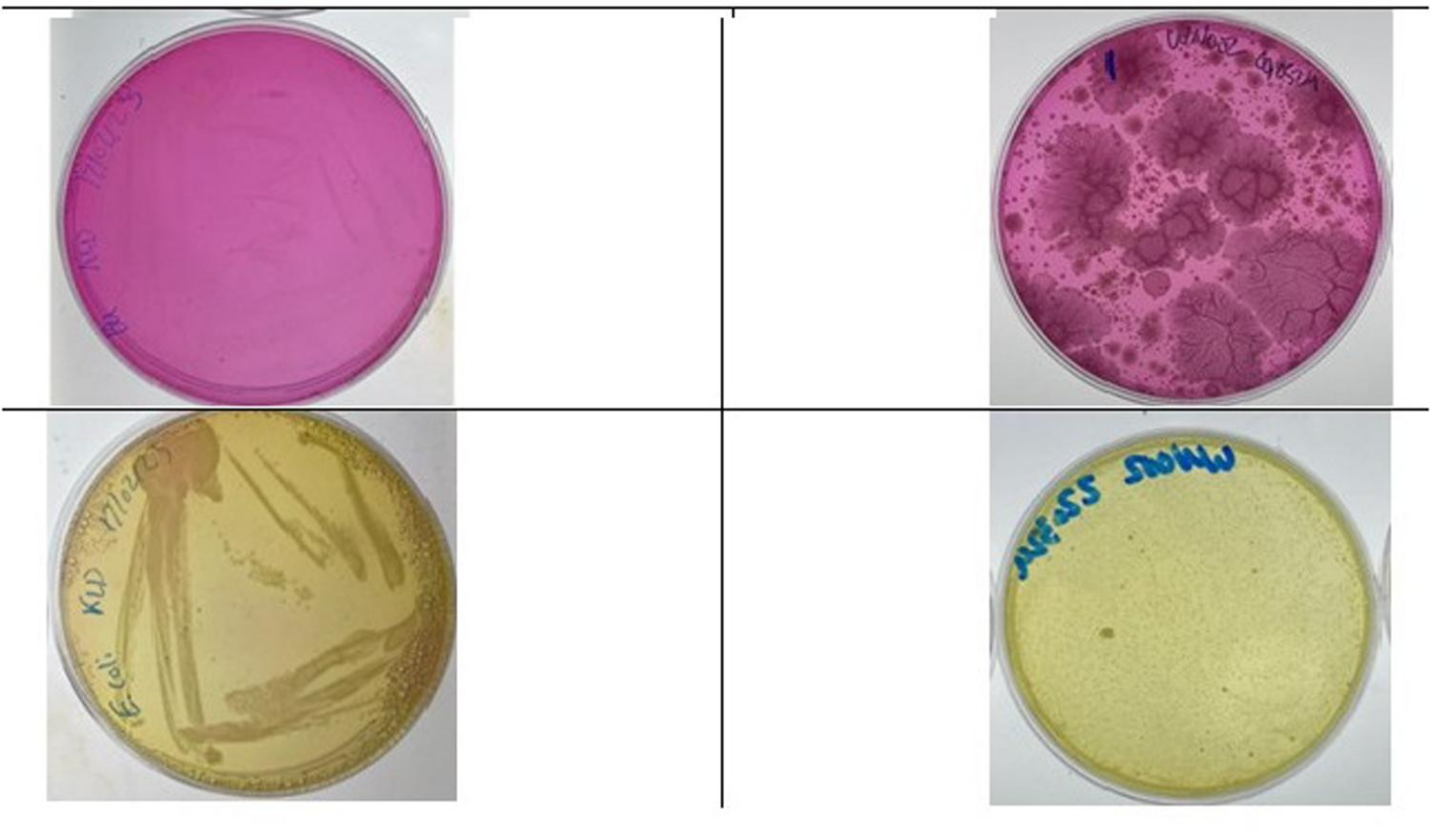
**(a)** Validation of phenol red urea agar showing urease-expressing Proteus mirabilis B4 (top); non-urease-expressing *Escherichia coli* (bottom). **(b)** Phenol red urea agar response to infant faecal sample containing urease-expressing bacteria (top) and non-urease-expressing bacteria (bottom).

Urease activity was quantified by thawing the Eppendorf tube, weighing out 250 mg of faecal material and re-suspending in phosphate-buffered saline (1 mL). Samples were vortexed for 10 s, sonicated in an Ultrawave sonicator for 1 min, and vortexed again for 10 s to ensure sample homogenisation. Homogenised samples (50 µL) were then spread on urea agar using a method adapted from Kohta et al. (11) and incubated at 37°C for 48 h. Urea agar contained the pH indicator, phenol red, which turns from yellow to bright pink at pH >7.4 (Figure 3), qualitatively indicating urease activity., as well as nutrients for bacterial growth and urea substrate for bacterial urease. Each faecal sample was cultured on three separate culture plates. A minimum of 80% plate area showing pink colour change was taken as the lower threshold for positive urease activity.

### 2.2 Patient and public involvement

Parents of young children were involved in preliminary studies used to validate the study protocol. The research question and outcome measures were developed in consultation with both nursery staff and parents. The study design involved discussions with nursery staff to ensure minimal disruption to nursery routines. Recruitment was conducted via posters displayed within the nursery and word-of-mouth discussions with parents arriving to collect their children. Posters of the study findings were created and displayed in the nursery on the advice of staff.

## 3 Results

### 3.1 Temporal association of nappy rash and faecal urease activity

**Figure 4:**
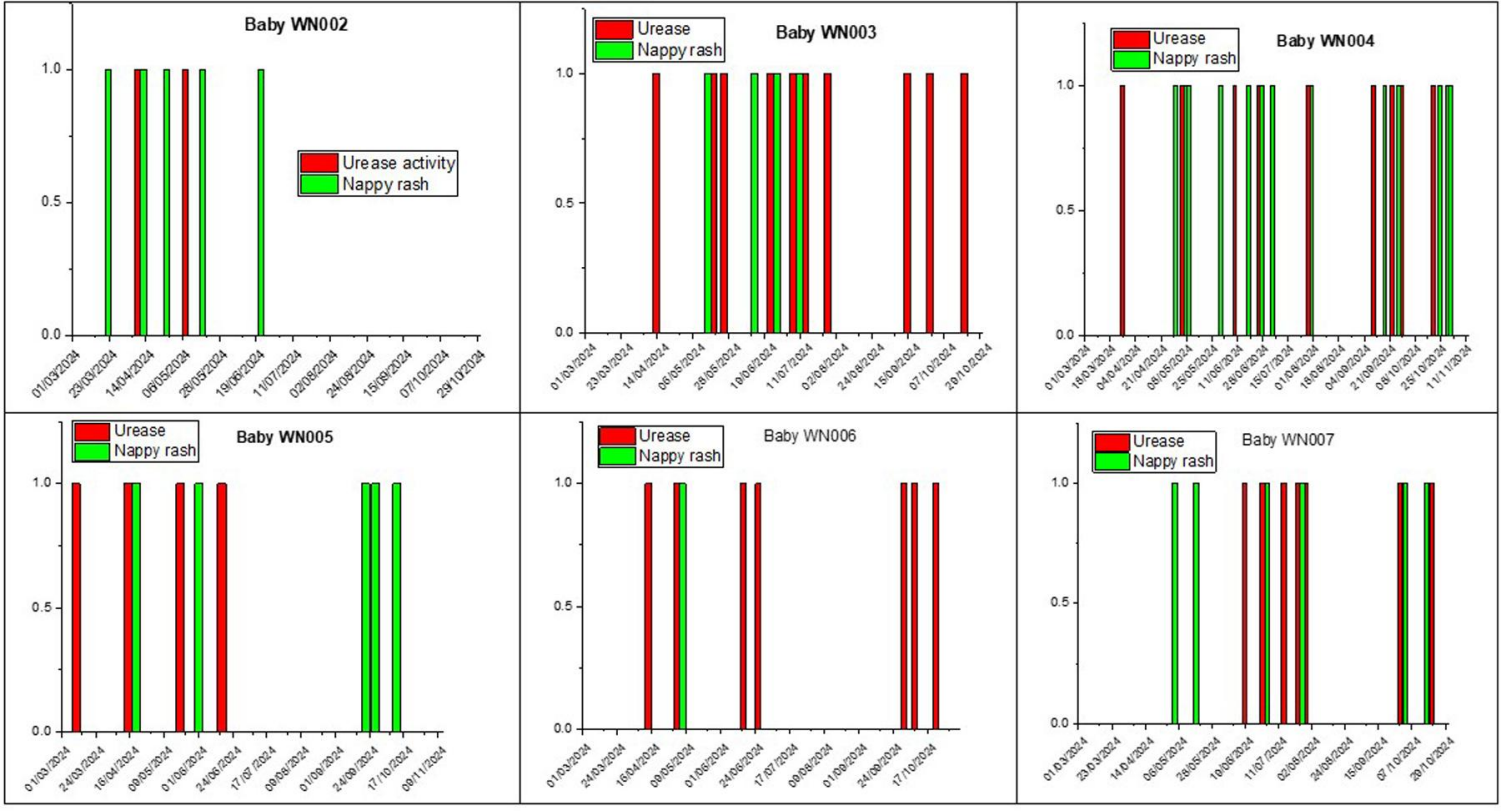
Temporal correlations between reported nappy (diaper) rash and urease-expressing faecal bacteria for the six babies in the study. Nappy rash incidence recorded (green bar) and measured faecal urease activity (red bar) plotted over the study duration. Full details provided in Supplementary Data, Table S1.

Nappy rash incidence and faecal urease expression were plotted as a function of study time (March–October 2025) for all six participants, as shown in Figure 4. Most babies demonstrated clustering of urease activity with nappy rash observation, rather than the random distribution one might expect if the two events were not correlated. Five of the six babies (WN002, WN003, WN004, WN005, and WN007) showed a clear apparent temporal association between faecal urease activity and observed nappy rash, although outliers were noted: WN002 had two nappy rash incidents but no correlating measured faecal urease; WN005 likewise had generally reasonable correlation until September 2024 when nappy rash was recorded on three occasions without measure urease; and WN006 had multiple incidents of high faecal urease but overall low nappy rash incidence. In most cases, elevated urease expression was observed in the week preceding or concurrent with the observed nappy rash episode. A full record of all recorded events for each child is provided in Supplementary Table S1.

### 3.2 Statistical analysis

Correlation between nappy rash and subsequent measurement of faecal urease was defined as positive (chi-squared test) if rash was observed on babies' skin up to 10 days following measurement of urease in their faeces. The rationale is that there would be a time delay between changes in their faecal microbiome and the rash being observed. The time window of ten days was chosen to allow nappies to be collected and skin health recorded, given that most babies were not attending nursery 5 days per week, ensuring time for skin exposed to urease to develop visible nappy rash.

The reports of nappy rash and urease activity (yes/no) were both non-parametric, so correlation was quantified by chi-squared analysis using GraphPad. The results grid is shown in Table 1, with positive correlation in green and negative in red.

**Table 1:** Results from all measurements (individually reported nappy rash and faecal urease of same baby at same time) of the six babies in the study with correlation between reported nappy rash and urease-positive faecal bacteria and vice versa.

	Observed nappy rash	Normal skin
Urease-positive faecal bacteria	21	23
Urease-negative faecal bacteria	14	76

Chi-square analysis performed using GraphPad Prism (https://www.graphpad.com/quickcalcs/chisquared1/) without Yates' correction yielded a χ² value of 14.227 with 1 degree of freedom and a two-tailed P value of 0.0002. This demonstrated a highly statistically significant association between faecal urease activity and the presence of nappy rash (and vice versa) was found to be extremely statistically significant.

## 4 Discussion

The results of this study suggest that there is temporal relationship between faecal urease and observed nappy rash. This can be mechanistically understood in terms of direct ammonia damage to the skin from the urease-expressing bacteria, consist with both Cooke and Keith's hypothesis from 1927 and more recent work showing how ammonia on skin directly damages the stratum corneum barrier function (3, 5). Ammonia is a weak base, which increases skin pH, promoting the growth of pathogenic bacteria and enhancing the activity of skin-degrading enzymes, thereby performing a dual role in nappy rash pathogenesis. Urease-expressing bacteria in faeces were detected only intermittently. Indeed, the correlation between children's healthy skin and the *absence* of measurable urease in their faeces was also significant.

Urease is an enzyme expressed by several enteric bacterial species, including *Proteus mirabilis.* It is reasonable to assume that many of the skin-dwelling bacteria found in the nappy area of infants have an enteric origin, creating the possibility of a future approach for preventing nappy rash by inhibiting bacterial urease and/or urease-expressing bacteria. This might be achieved in various ways: alteration of the infant gut microbiome by dietary modification—for example, by supplementation of infant formula with probiotics; direct topical application of urease inhibitors either as a cream or by modification of the skin-facing top sheet of nappies; and application of current topical nappy rash creams that work primarily via emollient and skin barrier effects, and in some cases exhibit a weak antimicrobial effect from zinc oxide.

Inhibiting urease may have multiple effects: (1) By reducing ammonia expression and skin ammonia concentration, stratum corneum integrity can be better maintained, reducing susceptibility to damage from hyper-hydration and friction. (2) Reducing ammonia concentration will ensure that skin pH can be maintained at healthy levels around pH 5.5 and crucially below pH 7, the level at which many skin-damaging faecal enzymes become much more active. (3) High pH (> pH 8) is known to change the morphology of commensal yeasts such as *Candida albicans*, altering from a relatively harmless spherical morphology to a hyphae-growing morphology, which is known to be capable of penetrating the skin epithelium (12).

Although this study demonstrated good statistical correlation between measured faecal urease and nappy rash incidence, and vice versa, there were significant outliers. These discrepancies likely relate to variations in children's skin susceptibility to ammonia as well as insufficient granularity in data collection. Moreover, differences in skin treatment and care regimes outside the nursery environment were not recorded, which may have contributed to different outcomes for children following skin exposure to urease/ammonia.

## 5 Conclusions

This study has some limitations. The most significant is the small sample size, which prevented the study from being statistically powered. Diagnosis of nappy rash was subjectively made by nursery staff. This was not a clinical environment, and it is likely that there was some variability in diagnosis based on staff experience and pre-conceived notions of the presentation of nappy rash. Ideally, clear photographs of the babies' nappy area would have been taken, with anonymous scoring of skin condition by third parties, but this would have made receiving parental consent more difficult. The differential diagnosis of nappy rash from other dermatological conditions such as atopic dermatitis and psoriasis has been discussed by Fölster-Holst (13). It is suspected that there may have been some over-reporting of nappy rash by nursery staff (“if you look for a condition, you find it”), but this is very difficult to control without a more rigorous diagnostic criteria using photographs assessed by third parties (14).

Another limitation was the binary on/off nature of the urease detection assay. The urea agar plate method was easy to use and gave clear results, but data were inherently qualitative. In principle, a more quantitative assay could have been used, but this would have made analysis considerably more complex. A pilot study using this approach showed very high variability in results, probably due to the difficulty in standardising faecal samples.

Despite these limitations, this study is the first, to our knowledge, to demonstrate a correlation between faecal urease and nappy rash incidence, as well as a correlation between the absence of faecal urease and no nappy rash. This suggests that urease may have a role to play in the onset of nappy rash. Further investigation to identify the specific faecal bacterial taxa responsible for these observations may improve understanding of the role of urease in nappy rash. Accordingly, 16S rRNA sequencing of faecal microbiota is currently underway, and the findings will be reported in due course.

## Data Availability

The original contributions presented in the study are included in the article/Supplementary Material, further inquiries can be directed to the corresponding author.
